# Star-Shaped Poly(furfuryl glycidyl ether)-Block-Poly(glyceryl glycerol ether) as an Efficient Agent for the Enhancement of Nifuratel Solubility and for the Formation of Injectable and Self-Healable Hydrogel Platforms for the Gynaecological Therapies

**DOI:** 10.3390/ijms22168386

**Published:** 2021-08-04

**Authors:** Piotr Ziemczonek, Monika Gosecka, Mateusz Gosecki, Monika Marcinkowska, Anna Janaszewska, Barbara Klajnert-Maculewicz

**Affiliations:** 1 Centre of Molecular and Macromolecular Studies, Polymer Division, Polish Academy of Sciences, Sienkiewicza 112, 90-363 Lodz, Poland; piotziem@cbmm.lodz.pl (P.Z.); gosecki@cbmm.lodz.pl (M.G.); 2 Department of General Biophysics, Faculty of Biology and Environmental Protection, University of Lodz, 141/143 Pomorska Street, 90-236 Lodz, Poland; monika.marcinkowska@biol.uni.lodz.pl (M.M.); anna.janaszewska@biol.uni.lodz.pl (A.J.); barbara.klajnert@biol.uni.lodz.pl (B.K.-M.)

**Keywords:** vulvovaginitis, candidiasis, Trichomonas infections, nifuratel, drug delivery system, hydrophobic drug, unimolecular copolymer micelle, poly(furfuryl glycidyl ether), poly(glyceryl glycerol ether)

## Abstract

In this paper, we present novel well-defined unimolecular micelles constructed a on poly(furfuryl glycidyl ether) core and highly hydrophilic poly(glyceryl glycerol ether) shell, PFGE-b-PGGE. The copolymer was synthesized via anionic ring-opening polymerization of furfuryl glycidyl ether and (1,2-isopropylidene glyceryl) glycidyl ether, respectively. MTT assay revealed that the copolymer is non-cytotoxic against human cervical cancer endothelial (HeLa) cells. The copolymer thanks to furan moieties in its core is capable of encapsulation of nifuratel, a hydrophobic nitrofuran derivative, which is a drug applied in the gynaecology therapies that shows a broad antimicroorganism spectrum. The study shows high loading capacity of the copolymer, i.e., 146 mg of nifuratel per 1 g of copolymer. The load unimolecular micelles were characterized using DLS and TEM microscopy and compared with the reference glyceryl glycerol ether homopolymer sample. The presence of numerous 1,2-diol moieties in the shell of PFGE-b-PGG macromolecules enabled the formation of reversible cross-links with 2-acrylamidephenylboronic acid-based polyacrylamide. The obtained hydrogels were both injectable and self-healable, which was confirmed with a rheological study.

## 1. Introduction

Vulvovaginitis is one of the most frequent gynaecological diseases [1,2]. The most common types of the vulvovaginal infections include vulvovaginal candidiasis, trichomonal vaginitis, and bacterial vaginosis [3]. In 2019, between 5% and 70% of all women worldwide were affected with bacterial vaginosis [4]. Vaginal candidiasis belongs to the most frequent *Candida* infections [5]. Almost 75% of women suffer from this disease at least once in their life [5]. In addition, some patients are infected with multiple pathogens simultaneously. Gynaecological treatment mainly relies on vaginal suppository administration. Unfortunately, such therapy is not effective, as suppository content is very often released in an uncontrolled manner. In fact, the amount of absorbed drug, as well as which part of the tissue is drug-exposed, is often unknown. It imposes frequent drug administration, up to several times a day, which is highly uncomfortable for patients, especially those that are professionally active. The lack of control over drug delivery may lead to disease reoccurrence [6] and thus the development of drug resistance. Inadequate treatment leads not only to chronic inflammation, but also to miscarriage or even infertility.

Conventional vaginal suppositories do not fulfil their function as a result of poor adhesive properties and short retention time [7]. To address these challenges, hydrogel formulations are designed to improve drug delivery [8,9]. It results from their compatibility with aqueous environments, biocompatibility, high porosity, and in addition, their ability for controlled drug delivery [10]. Hydrogels can provide a spatial and temporal control over the release of various therapeutic agents, including both small molecules and macromolecular drugs. They possess modulable physical properties and the capability to protect labile drugs from degradation controlling their release. The porous structure of hydrogels enables loading of drugs into the gel matrix and afterwards drug release at a rate contingent on the diffusion coefficient of the molecule through the gel network. The real benefits of hydrogels used for drug delivery are that they have a pharmacokinetic nature, and are associated with leisurely drug elution while maintaining a high local concentration of drug in the tissue over an extended period [11].

The most suitable hydrogel system for the gynaecological treatment, however, should exhibit shape adjustment to the covered surface, which would facilitate the formation of continuous coverage of afflicted areas and would prolong the retention time of pharmacological formulation to be not easily removed by the mechanism of the self-cleaning process. Traditional hydrogels, i.e., based on irreversible covalent cross-links, fail, as the cross-linking process defines their shape and the proper fit to the tissue surface is impossible. On the contrary, the dynamic hydrogel systems constructed on adaptable linkages, i.e., reversible covalent [12,13,14] or supramolecular bonds, [15,16,17,18], which are broken and reformed in a reversible manner without the usage of external triggers, assures besides the injectability, also the formation of a continuous hydrogel layer on the infected site.

The usage of hydrogel formulations in gynaecological therapies, however, is not straightforward. The main problem results from the hydrophilic character of hydrogels, while most medicines applied in the gynaecological treatment are hydrophobic, and thus are poorly soluble in a hydrogel matrix. The controlled administration of water-insoluble drugs is of great importance as it results in the reduction of the amount of administered drug, and by consequence it limits drug-related toxicity and side-effects. This can be achieved by the use of hydrogels built from block amphiphilic copolymers [19] prone to micelle formation. From one side, hydrophobic segments in the inner part of micelle improve the solubility of poorly soluble drugs in water. From the other side, hydrophilic moieties assure solubility in water for the drug-loaded macromolecular constructions. The encapsulation of drugs in micelles can eliminate drug side effects and provide protection of drug molecules against possible degradation [20]. The disadvantage of standard block copolymer micelles is related to the fact of micelle formation only above the critical micellar concentration. Due to this fact, the construction of the hydrogel platform based on these building blocks is inconvenient. What is more advantageous is the usage of unimolecular polymer micelles, i.e., polymer structures, composed of covalently bound amphiphilic chains, that mimic the construction of polymer micelles, which are, however, intrinsically stable, and thus they preserve their structure regardless of the concentration [21]. Among unimolecular polymer micelles, dendrimers, [22,23] hyperbranched polymers, [24,25] and star-shaped polymers [26] can be distinguished.

In the case of hydrophobic drugs applied in the gynaecological treatment of vagina infections, medicines of a broad spectrum of action deserve special attention, [27,28,29,30,31,32] such as nifuratel, a nitrofuran derivative belonging to the furazolidone class [30]. It displays not only antibacterial properties [31] (against both Gram-negative and Gram-positive organisms) [33] and anti-protozoan action against Trichomonas vaginalis, but also it inhibits the growth of *Candida albicans* [31,32]. Nifuratel has been reported to possess anticancer properties against cancer cells. The results show that nifuratel inhibits the proliferation, induces apoptosis and blocks the interleukin-6 (IL-6)-mediated activation of the STAT3 signalling pathway. This is considered to be one of the possible mechanisms by which the expression of proapoptotic proteins is upregulated and the proliferation of gastric cancer cells is inhibited [34,35].

There are only few reports focused on the construction of systems suitable for the enhancement of nifuratel solubility. These include a liposome system [36], and commercially available nifuratel-based soft capsule suppository *Macmirror Complex* [37]. The latter platform is composed of a substantial amount of the drug (500 mg), the dose of which is strictly related to the relationship between drug concentration and the therapeutic efficacy [28]. Lower drug content resulted in worse therapeutic effect [28]. Generally, drugs of limited water solubility display slow drug absorption and often require the usage of high doses in order to attain a therapeutic concentration.

To improve hydrophobic drug bioavailability, various carriers have been developed, among which unimolecular micelles appear to address the issues related to the vaginal therapies. So far, there are no advanced polymer systems adapted to the nifuratel solubilization. In this article, we demonstrate a convenient synthesis route of unimolecular micelles composed of hydrophobic polymer segment equipped with furfuryl units poly(furfuryl glycidyl ether) core and hydrophilic shell of poly(glyceryl glycerol ether). Until now, star-shaped amphiphilic unimolecular micelles based on a furan-based core and diol-enriched shell with the usage of epoxide-based comonomers has not benne reported. Generally, furfuryl glycidyl ether was applied to form diblock [38] and triblock terpolymer [39] with comonomers such as ethylene oxide and allyl glycidyl ether. Furan-containing polymers found application in thermoresponsive or photothermoresponsive hydrogel systems for controlled drug release or to form carriers for covalent immobilization of drug molecules, which upon the external stimulus, can be cleaved [40]. Such materials are formed based on Diels–Alder chemistry. The similar synthetic approach is used for the generation of self-healable cross-linked coatings based on polymers equipped with furan moieties.

In this work, furan-rich fragments are designed to increase solubility of nifuratel, whereas the particle shell enriched with numerous vicinal diol moieties is responsible for assuring the macromolecule solubility in water and enables reversible cross-linking with boronic acid moieties to obtain hydrogel. The idea of furan moieties exploitation to improve nifuratel solubility resulted from the enhancement effect of doxorubicin solubility upon the prior macromolecule functionalization via covalent immobilization with that drug [41,42] The similarity of molecular composition of both furfuryl glycidyl ether and nifuratel encouraged us to use this approach.

The effect of furan moieties on the solubilization of nifuratel in water was investigated using ^1^H NMR spectroscopy, DLS and TEM microscopy. Moreover, the rheological properties such as viscoelastic behaviour, injectability, and self-healing properties of hydrogel systems composed of unimolecular micelles cross-linked with boronic acid-based polyacrylamide were studied in view of the vaginal therapies. To test the biological effect, we have chosen HeLa cells as our experimental model. This cell line is routinely used to screen anticancer properties of different drugs, esp. used in gynaecological treatments.

## 2. Results and Discussion

### 2.1. Synthesis of Star-Shaped Amphiphilic Block Copolymer

**Figure 1:**
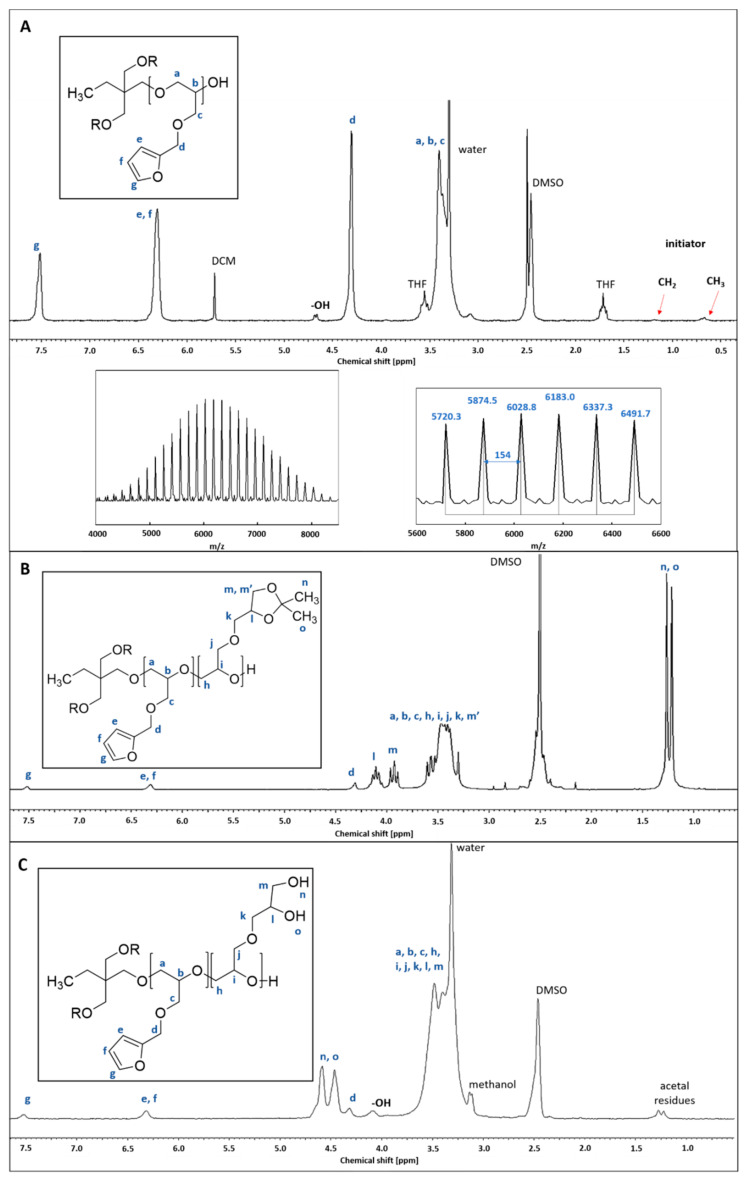
(**A**) ^1^H NMR spectrum recorded in DMSO-d_6_ and MALDI-TOF mass spectra of poly(furfuryl glycidyl ether), PFGE; (**B**) ^1^H NMR spectrum of poly(furfuryl glycidyl ether)-b-poly((d,l-1,2-isopropylidene glyceryl) glycidyl ether), PFGE-b-PIGG recorded in DMSO-d_6_; (**C**) ^1^H NMR spectrum of poly(furfuryl glycidyl ether)-b-poly(glyceryl glycerol ether), PFGE-b-PGGE recorded in DMSO-d_6_. Blue letters correspond to appropriate protons.

The presence of a nitrofuran moiety in the nifuratel molecule inspired us to design unimolecular copolymer micelles bearing furfuryl moieties in the core, applying the basic rule: “like dissolves like”, i.e., substances with similar chemical characteristics will dissolve in each other. Therefore, at first, we built a furan-based hydrophobic core applying ring-opening anionic polymerization of furfuryl glycidyl ether initiated with 1,1,1-tris(hydroxymethyl)propane, which hydroxyl groups were partially conversed into sodium alcoholates in the reaction with NaH. The synthesized homopolymer was a three-armed polyether carrying a furfuryl pendant group in each constitutional unit. The formation of this polymer was confirmed based on ^1^H NMR spectroscopy, GPC and MALDI-TOF. The disappearance of signals corresponding to protons of epoxide ring (2.35–2.75 ppm), Figure S1, along with the appearance of protons signals of the ether backbone at 3.10–3.60 ppm (Figure 1A), indicated the occurrence of ring-opening polymerization. In addition, the integration of protons **e**, **f**, **g** of the furfuryl ring correlates with the integration of protons **d**, which excludes the occurrence of uncontrolled cross-linking with the participation of furfuryl moieties. In the MALDI-TOF spectrum, the main population was ascribed to the desired PFGE homopolymer (Figure 1A). The *m*/*z* of the signals corresponds to consecutive macromolecules with CH_3_C(CH_2_O)_3_, 2H end groups coming from an initiator and terminal OH groups, respectively, and K^+^ counterion. The signals are separated from each other by 154, which corresponds to the molecular weight of an FGE unit. The highest peak at *m*/*z* equals 6028.8, corresponding to a macromolecule composed of 38 repeating units. The distribution observed in the MALDI-TOF spectrum corresponds well to molecular weight determined with ^1^H NMR end groups analysis ($\bar{DP}n$ = 39). GPC confirmed that the polymerization of furfuryl glycidyl ether was controlled under the conditions applied as the product molecular weight distribution was narrow M_w_/M_n_ = 1.11.

**Scheme 1:**
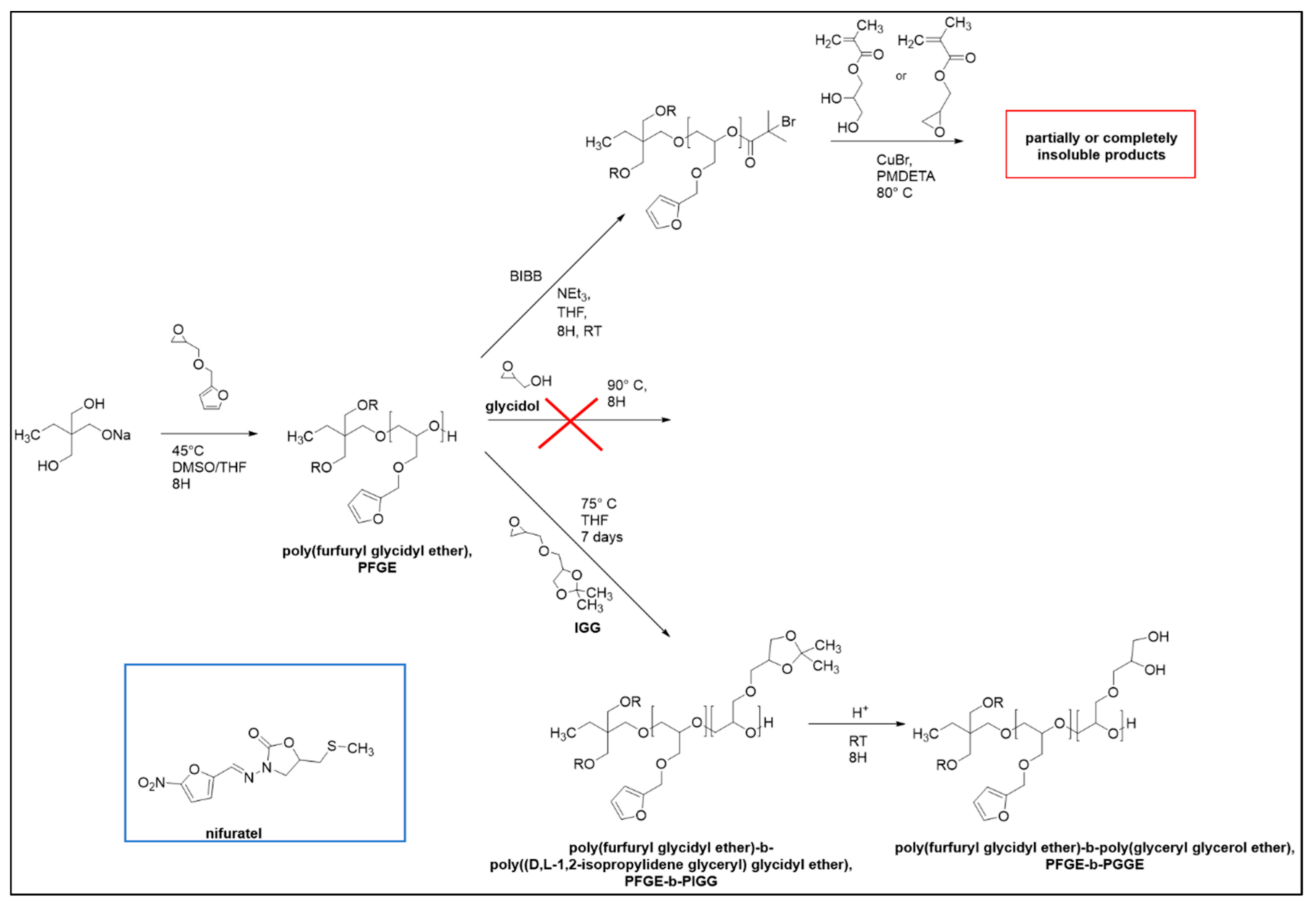
Illustration of synthetic routes of amphiphilic copolymer formation and molecular structure of nifuratel.

The synthesized poly(fufuryl glycidyl ether) was applied as a macroinitiator to obtain the block copolymer structure. Due to the planned three-armed copolymer with hydrophobic core and hydrophilic shell, we established that the length of the hydrophilic shell-forming segments should be approximately ten times longer than the core segments to prevent the macromolecule aggregation and assure a copolymer complete solubilization. To synthesize the block copolymer structure with a hydrophilic shell, which would assure the cross-linking with boronic acid moieties, several strategies were tested (Scheme 1). Initially, we used controlled radical polymerization ATRP of methacrylate comonomers such as glycerol methacrylate or glycidyl methacrylate, bearing or being a precursor to diol groups, respectively, upon prior functionalization of PFGE end hydroxyl groups with α-bromoisobutyryl bromide. The polymerization process, however, displayed limited control over molecular weight or obtained products were partially or completely insoluble. The uncontrolled cross-linking processes possibly resulted from the presence methacrylate dimer forms, or furan rings in the macroinitiator [43].

We have also used poly(furfuryl glycidyl ether) as a macroinitiator of the anionic polymerization of glycidol after prior conversion of hydroxyl groups into sodium alcoholates. This approach, however, also did not succeed, as the final product was composed of two homopolymers, i.e., unreacted macroinitiator and polyglycidol of hyperbranched topology. We assumed that the significant difference in the nature of both species, i.e., hydrophilic comonomer and hydrophobic polymer, was responsible for the failure of the copolymerization, as the PFGE end alkoxide active sites in the polymerization are not well accessible for comonomer molecules. Therefore, instead of the glycidol, we applied a hydrophobic comonomer d,l-1,2-isopropylidene glyceryl glycidyl ether, IGG, i.e., glyceryl glycerol ether with ketal protected diol group. Its copolymerization conducted from a furan-based macroinitiator at 75 °C in tetrahydrofuran resulted in the formation of core-shell structure composed of three-armed poly(furfuryl glycidyl ether)-block-poly((d,l-1,2-isopropylidene glyceryl) glycidyl ether), PFGE-b-PIGG. The decay of epoxide protons of IGG (Figure S2) simultaneously with the appearance of the signal corresponding to the polyether backbone (Figure 1B) confirmed the process of copolymerization. GPC (Table 1) and ^1^H DOSY NMR (Figure S3) confirmed the formation of one polymer population. PFGE-PIGG was soluble in methanol, CH_2_Cl_2_, THF, DMF and DMSO. The hydrolytic cleavage of the acetal protecting groups in the presence of 10 moL% aqueous solution of HCl leads to the almost complete release of glycerol moieties, i.e., 96.5 moL%, and formation of the amphiphilic copolymer poly(furfuryl glycidyl ether)-block-poly(glyceryl glycerol ether), PFGE-b-PGGE, which was confirmed with ^1^H NMR spectroscopy (Figure 1C). The final product was soluble in water, methanol, ethanol, DMF and DMSO.

In spite of encountered synthetic problems with successful grafting of the second block from poly(furfuryl glycidyl ether)-based macroinitiator, we elaborated a convenient route to the formation of star-shaped amphiphilic copolymers with a furan-based hydrophobic core and a highly hydrophilic shell enriched with numerous diol functional groups applying ring-opening anionic copolymerization. The presence of multiple diol moieties opens the variety of modification strategies of the peripheral area of the macromolecules.

### 2.2. Cytotoxicity

**Figure 2:**
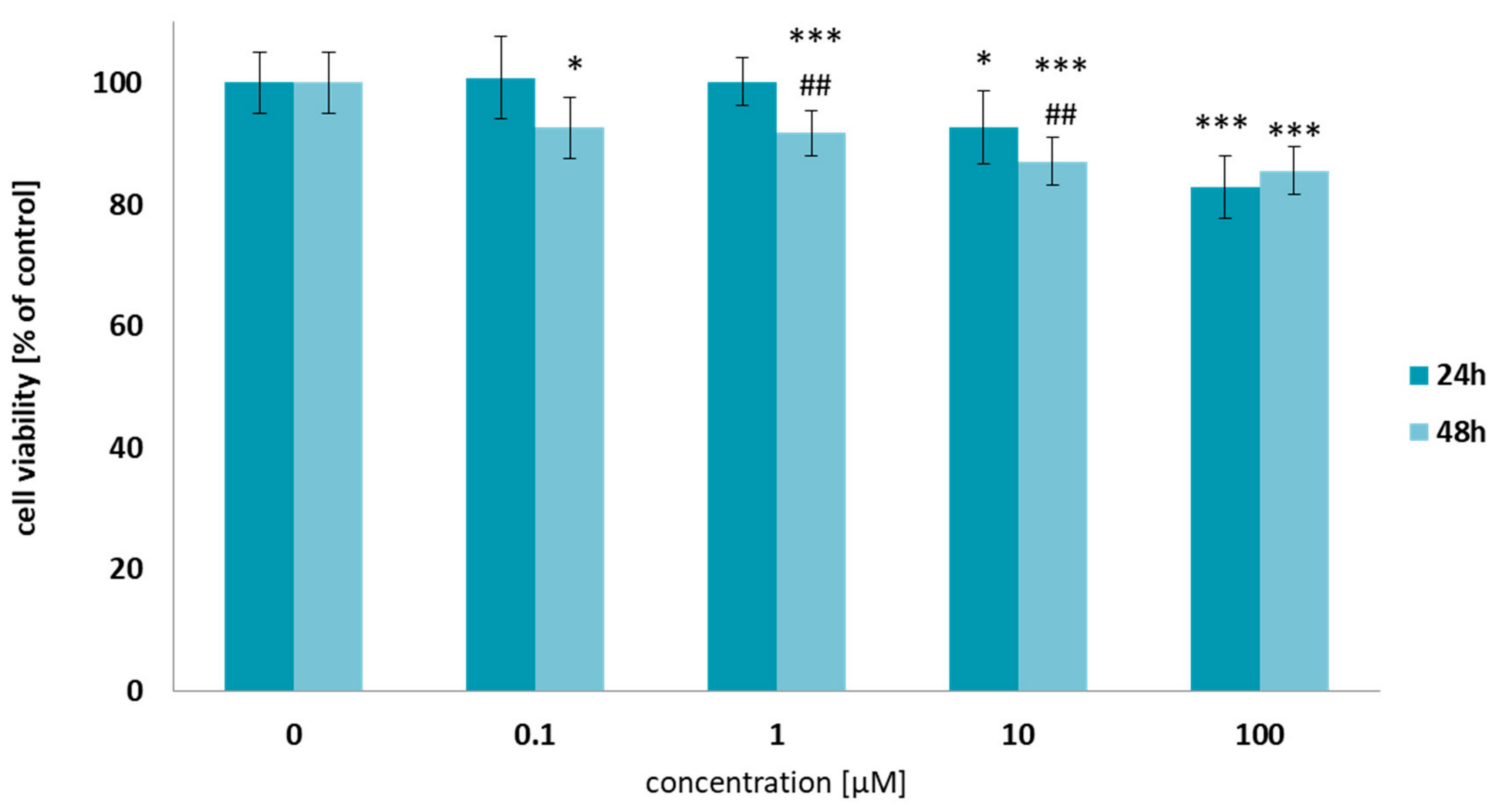
The influence of PFGE-b-PGGE on viability of human cervical cancer endothelial (HeLa) cells after 24 h (dark blue) and 48 h (light blue) incubation at 310 K. Data are presented as a percentage of control (untreated cells) ± standard deviation (SD). * *p* < 0.05, *** *p* < 0.001 statistically significant differences in comparison to control cells; **##** *p* < 0.01 statistically significant differences between probes incubated with the same copolymer concentration after treatment of HeLa cells at 24 and 48 h.

The assay was conducted to assess the cytotoxicity of the synthesized amphiphilic copolymer PFGE-b-PGGE. It was evaluated based on the viability of human cervical cancer endothelial (HeLa) cells after 24 and 48 h incubation at 310 K. As shown in Figure 2, even for the highest used concentration of 100 µM, no decrease in cell viability below 80% was observed after 48 h incubation with the polymer. The absence of cytotoxicity of such high concentration is important in view of hydrogel formulations. The use of the amphiphilic copolymer poly (furfuryl glycidyl ether)-block-poly (glyceryl glycerol), water-soluble PFGE-b-PGG formation made it possible to avoid the use of DMSO, which, with a highly polar domain characterized by a sulfinyl group and two apolar methyl groups, is very toxic to eukaryotic cells.

### 2.3. Nifuratel Encapsulation

In the next step, we optimized the strategy of the efficient nifuratel encapsulation within the constructed PFGE-b-PGGE copolymer. For this goal, we exploited the method of solvent evaporation [44,45]. Other attempted methods, such as nanoprecipitation, were unsuccessful. Methanol was chosen as a good solvent of both formulation components, nifuratel and PFGE-b-PGGE, and due to the ease of its subsequent removal by evaporation. We prepared separate solutions of PFGE-b-PGGE and nifuratel, each one in methanol. Both solutions were combined and methanol was slowly evaporated. Then, the whole content was suspended in deionized water. The encapsulation efficiency (EE%), i.e., total amount of encapsulated drug vs. total amount of added drug was almost quantitative, approximately 90% up to 146 mg_(nifuratel)_/g_(FGE-b-PGGE)_.

At the maximum drug loading, approximately 43 molecules of nifuratel were encapsulated in the frame of one PFGE-b-PGGE macromolecule, which was determined based on ^1^H NMR spectra (Figures S4 and S5). The method of internal reference (DMF) additive was applied for the analysis. The known amount of DMF was introduced into the sample of nifuratel-enriched PFGE-b-PGGE, and integrations of DMF proton at 8.02 ppm, nifuratel protons at 7.15 ppm, and on FGE unit protons at 6.36 ppm were compared (Figure S5).

**Figure 3:**
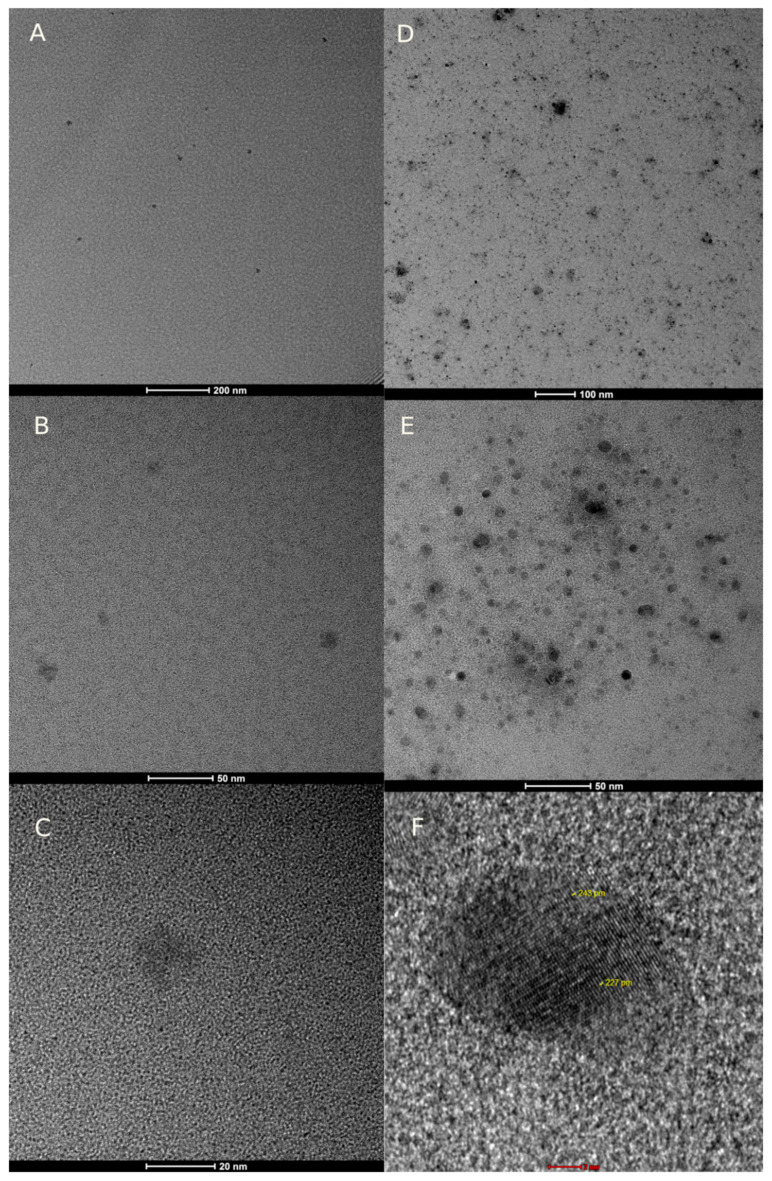
The representative TEM images of PFGE-b-PGGE macromolecules (**A**–**C**) and PFGE-b-PGGE macromolecules saturated with nifuratel (**D**–**F**).

**Figure 4:**
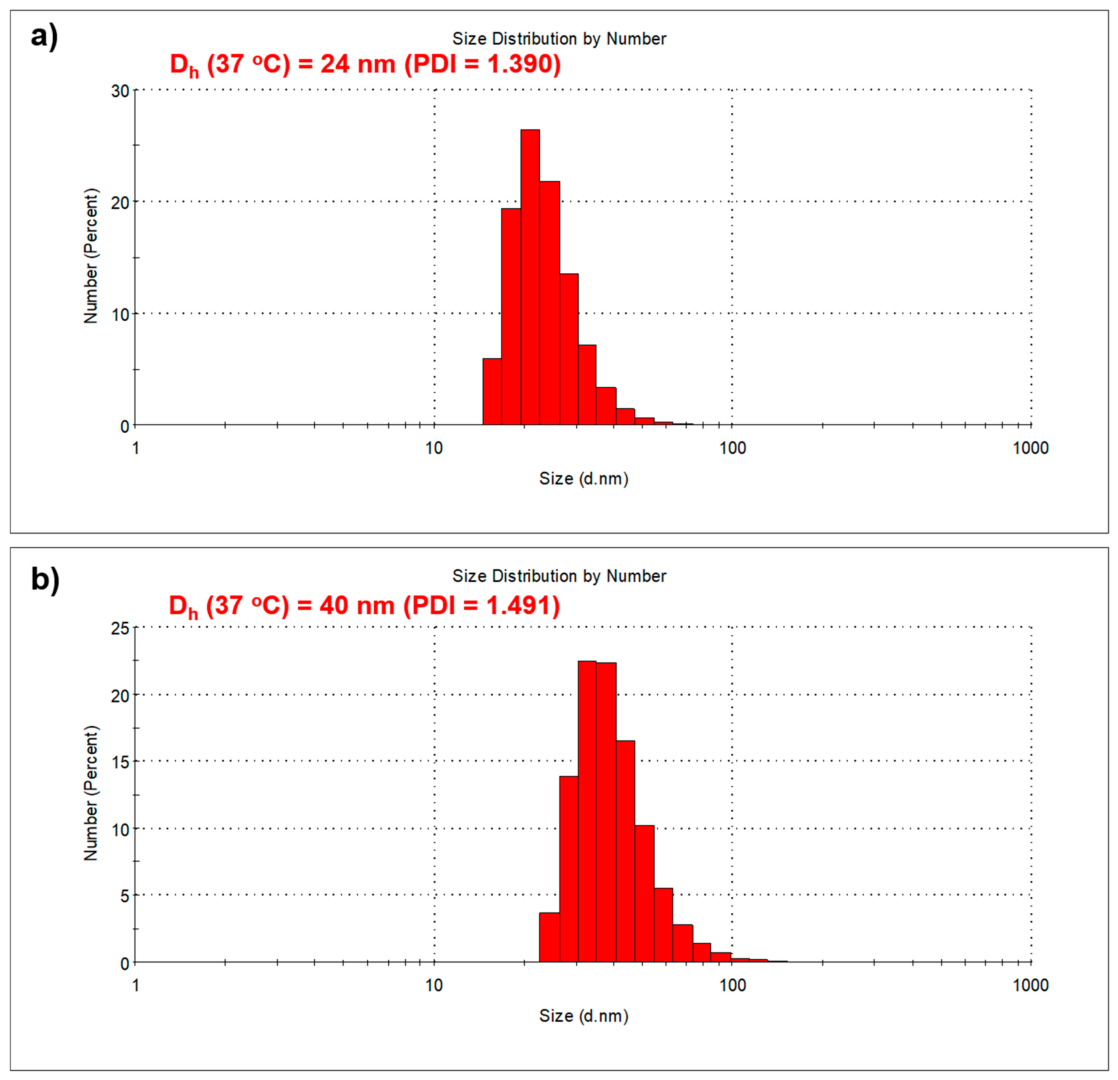
The DLS data of PFGE-b-PGGE macromolecules before (**a**) and after nifuratel encapsulation (**b**).

The structure of native PFGE-b-PGGE and their macromolecules upon saturation with nifuratel was investigated with DLS and TEM. The aqueous solution of nifuratel-enriched PFGE-b-PGGE was opalescent (Figure S6b), whereas any attempt to dissolve nifuratel in water resulted in a completely transparent solution that was free of nifuratel solution after filtration via syringe PTFE filter (0.45 µm), which was confirmed with ^1^H NMR in DMSO after a sample lyophilization. Generally, TEM images of neat PFGE-b-PGGE revealed the presence of nanosized spherical objects of average diameter 12.7 ± 2.1 nm (Figure 3A–C), whereas the hydrodynamic diameter determined at 37 °C based on DLS measurement was 24 nm (Figure 4). The higher value of particle hydrodynamic diameter resulted from the swollen state of macromolecules thanks to the highly hydrophilic character of the shell. Moreover, the dark spots on TEM images recorded at these conditions correspond to the PFGE-based hydrophobic core [46]. Upon drug encapsulation, the average hydrodynamic diameter of nanoparticles slightly increased to 40 nm. TEM also revealed crystalline domains in the frame of drug-loaded particle that were not visible in the neat copolymer images. These images support the successful nifuratel molecule encapsulation within the frame of the PFGE-b-PGGE macromolecules.

The control experiment performed for poly(glyceryl glycerol ether) homopolymer, PGGE (M_n_ = 21,000) before and after the process of drug encapsulation, analogous to that performed for copolymer, did not reveal any change of the object diameter and no crystalline domains were visible. In addition, the quantitative ^1^H NMR analysis revealed the encapsulation of approximately 0.6 moles of nifuratel per one PGGE macromolecule, and it can be concluded that nifuratel encapsulation in PGGE macromolecules is negligible. Moreover, these data undoubtedly show that a furan-rich copolymer core is a key factor of efficient nifuratel solubilization in the PFGE-b-PGGE system.

**Figure 5:**
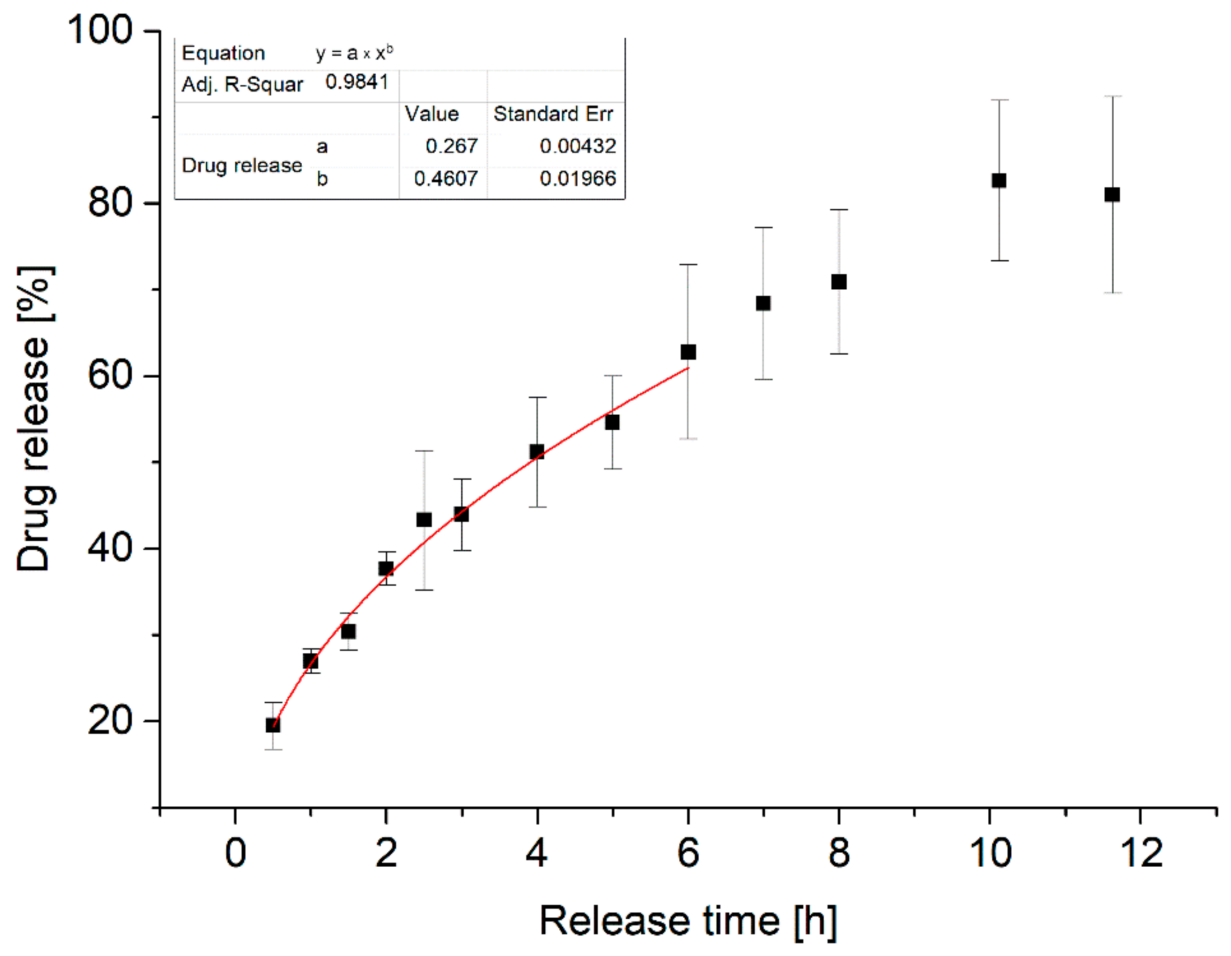
Nifuratel release profile from PFGE-b-PGGE. Red line shows fitting to the Korsmeyer–Peppas model.

For nifuratel-enriched PFGE-b-PGGE macromolecules, the concentration of released nifuratel was determined with UV-Vis spectroscopy in acetonitrile based on the calibration curve (Figure S7). The obtained in vitro release profile (Figure 5) shows the drug release from the PFGE-b-PGGE unimolecular micelles in approximately 12 h with only minor burst effect, approximately 20%, in first 30 min. The data, for up to 60% of total drug release, were fitted to a Korsmeyer–Peppas model with good correlation [47,48]. The determined *n* coefficient that characterizes the mechanism of drug release was is equal 0.46. The value indicates the diffusion-controlled release mechanism of nifuratel from the formulation.

### 2.4. The Formation of Hydrogel Systems and Their Rheological Properties

**Scheme 2:**
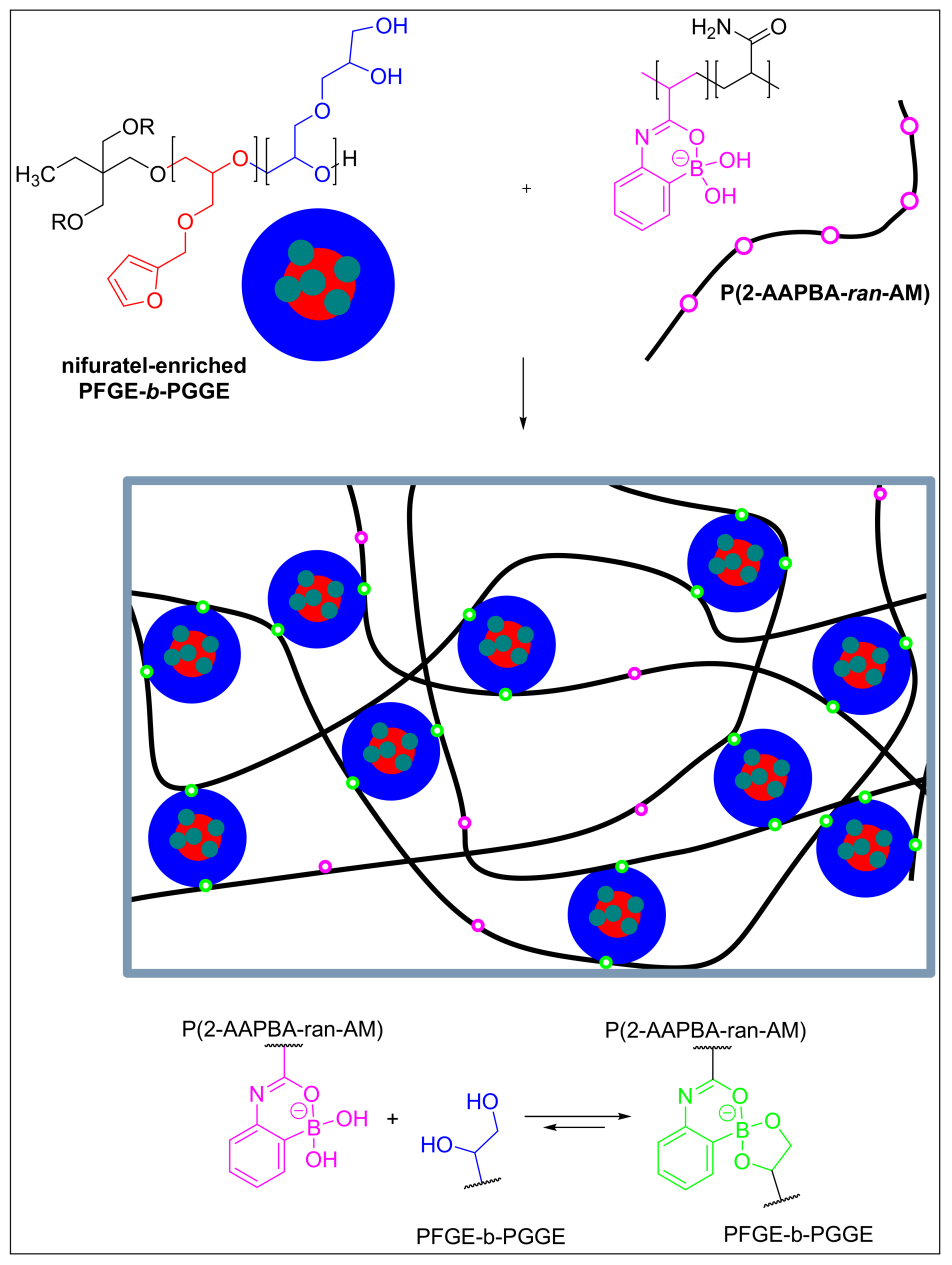
The illustration demonstrating the formation of reversible hydrogel based on nifuratel-enriched PFGE-b-PGGE cross-linked with 2-acrylamidephenylboronic acid-based polyacrylamide.

The construction of the copolymer shell composed of poly(glyceryl glycerol ether) was dictated with the presence of numerous 1,2-diol moieties, which are prone to form boronic esters in the reaction with boronic acids (Scheme 2). We verified the ability of star-shaped PGFE-b-PGGE macromolecules to form reversibly cross-linked hydrogel systems with polyacrylamide bearing 6 moL% of 2-acrylamidephenyl-boronic acid, 2-AAPBA constitutional units (Figure S8) at pH = 5.5, which corresponds to vagina pH. The usage of 2-AAPBA was dictated by its ability to cross-link not only at neutral pH, but also in acidic conditions [25], which is of great importance for the gynaecology application. Moreover, it has been proven that boronic acids display little toxicity on the human body [49,50]. For this aim, we prepared two hydrogel systems differing in the polymer total weight fraction, i.e., H1 and H2 composed of 23 and 18.5 wt% of dry weight, respectively (Table 2). The diol-to-boronic acid groups ratio in H1 and H2 hydrogel was 8.9 and 17.8, respectively.

**Figure 6:**
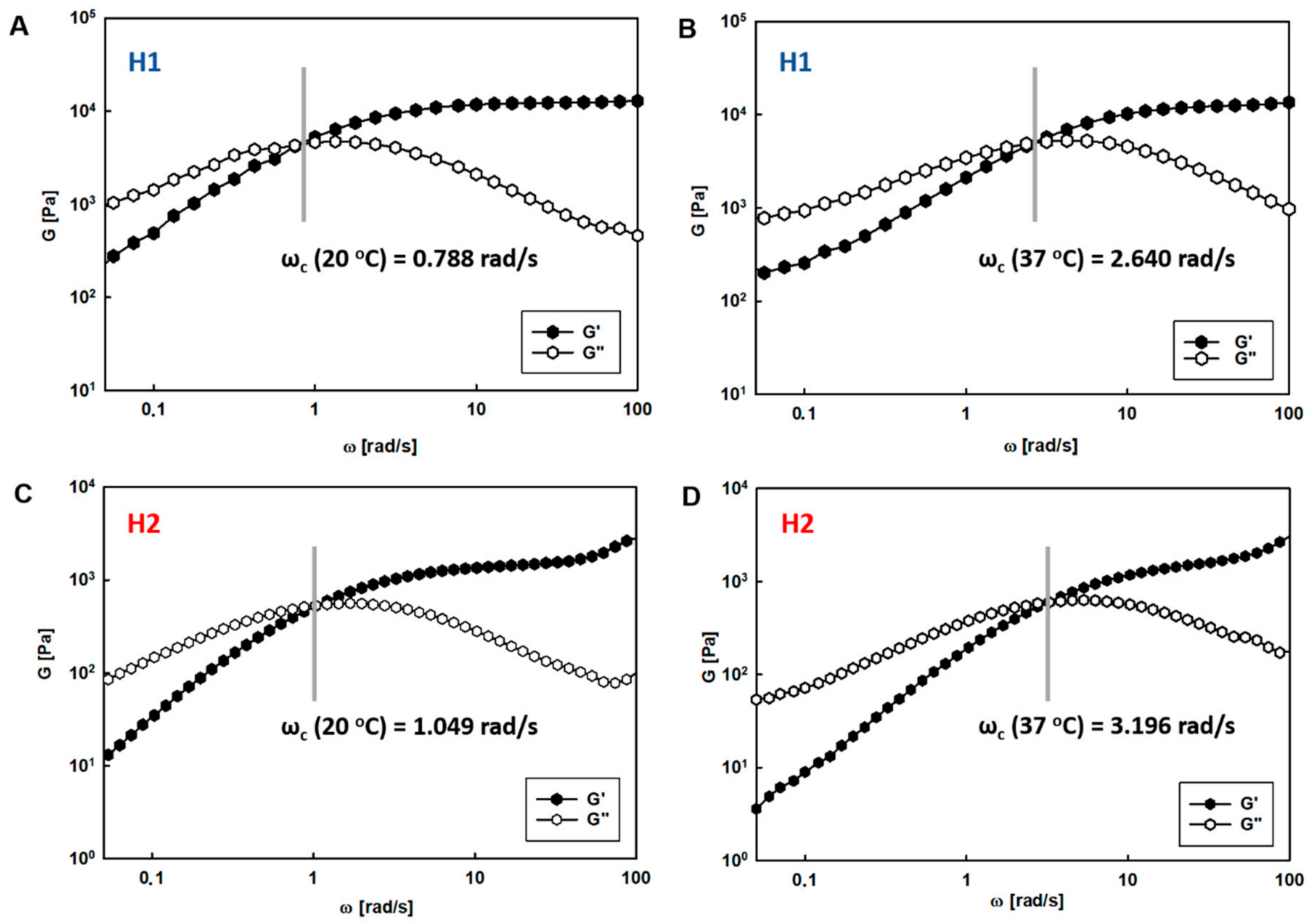
Frequency dependence of storage and loss moduli for hydrogel systems H1 and H2 composed of PFGE-b-PGGE cross-linked with 2-AAPBA-AM copolymer at pH = 5.5 recorded at 20 °C (**A**,**C**) and 37 °C (**B**,**D**), respectively.

Frequency sweep tests (Figure 6) performed for both hydrogels revealed that prepared systems display viscoelastic properties, and that the storage modulus does not cross the loss modulus at its maximum (ω_c_), which indicates that for the hydrogel formation, beside the boronic ester cross-links, some physical entanglements coming from both polymer components are responsible. The hydrogel composed of a higher fraction is stiffer in comparison to the less concentrated system, as the values of the storage modulus plateau, as the G’_max_ recorded at 20 and 37 °C for H1 is higher than that observed for H2. In addition, the increase of polymer content in the hydrogel resulted in the shift of ω_c_ toward lower values, i.e., from 1.05 to 0.79 rad/s at 20 °C, respectively, which corresponds to the longer lifetime of boron cross-links, and thus to the slower relaxation dynamics. It results from the inverse dependence of relaxation time (τ) and the crossover frequency: τ = (ω_c_)^−1^, where ω_c_ corresponds to the crossover frequency at which G′ = G′′ [51]. The variation of ω_c_ with temperature evidences the change in the association/dissociation rate of cross-links, which directly influences the rheological hydrogel properties. Generally, along with the gradual increase of temperature, the lifetime of boronic esters decreases, as the observed ω_c_ values became higher. It is noteworthy, that in the case of these systems, i.e., the star-shaped polyether, the shift of ω_c_ in the direction of higher values corresponding to the shorter effective lifetime of cross-links is not as evident as it was observed for hyperbranched polyglycidol at molecular weight, which is free of physical entanglements [25]. It shows that the fraction of physical entanglements in the star-shaped system at the given molecular weight are evidently higher than in hyperbranched polymers, and thus the investigated systems are less sensitive to temperature.

**Figure 7:**
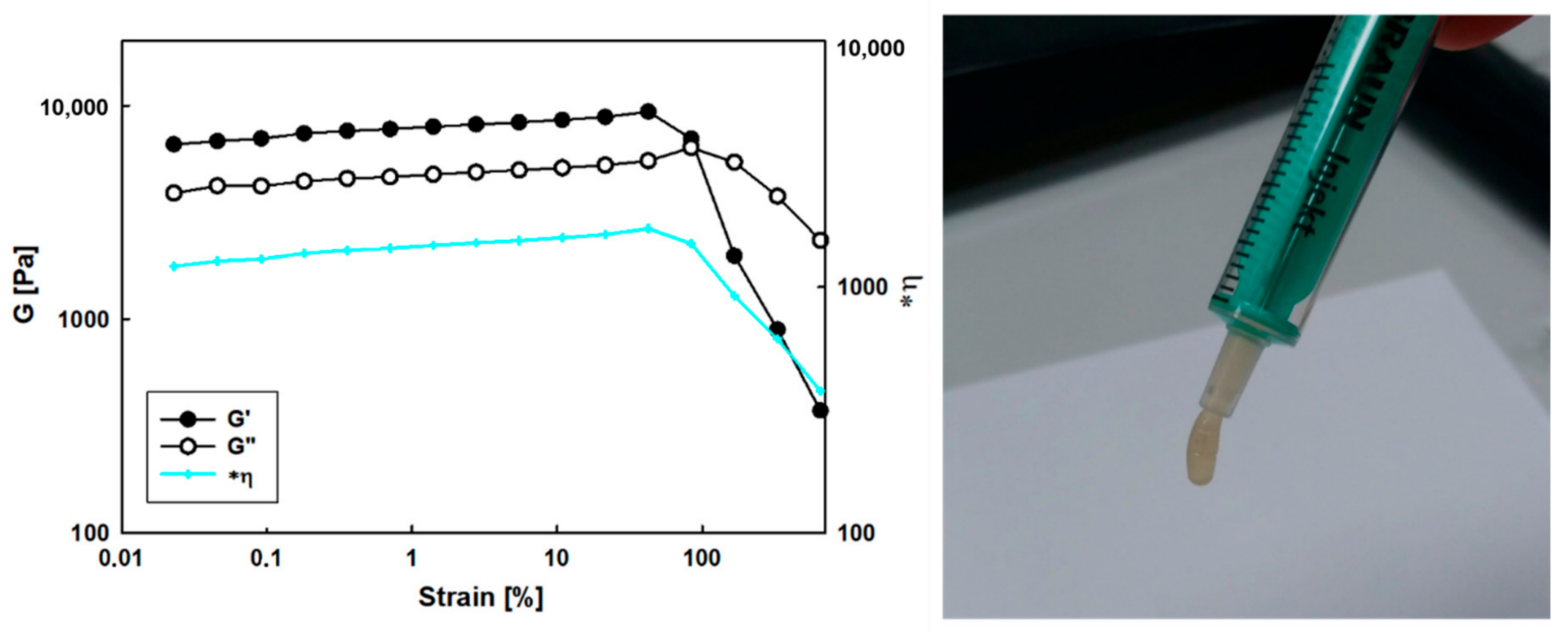
Strain sweep test recorded for hydrogel H1 demonstrating the hydrogel injectable properties at 37 °C (**left**). The hydrogel squeezing from the syringe at 25 °C (**right**).

The construction of the hydrogel systems as a platform for drug delivery in gynaecological therapies requires an analysis in view of the possibility of its incorporation into the vagina to the afflicted area. For this goal, we performed the strain sweep test at 37 °C to determine the injectability properties at the simulated conditions of the vagina. Generally, the yield stress (yield point), i.e., the lowest shear stress value above which a material behaves like a fluid, and below which the hydrogel is like a solid. For instance, the yield point determined for hydrogel H1 was 7.9 kPa, as the modulus G′ at this point decreased below its values in the whole examined linear viscoelasticity range (Figure 7). The relatively high yield point prevents the hydrogel from flowing under gravitation [52]. The gel, however, can be applied with a syringe on the inflicted area even at 25 °C (Figure 7).

**Figure 8:**
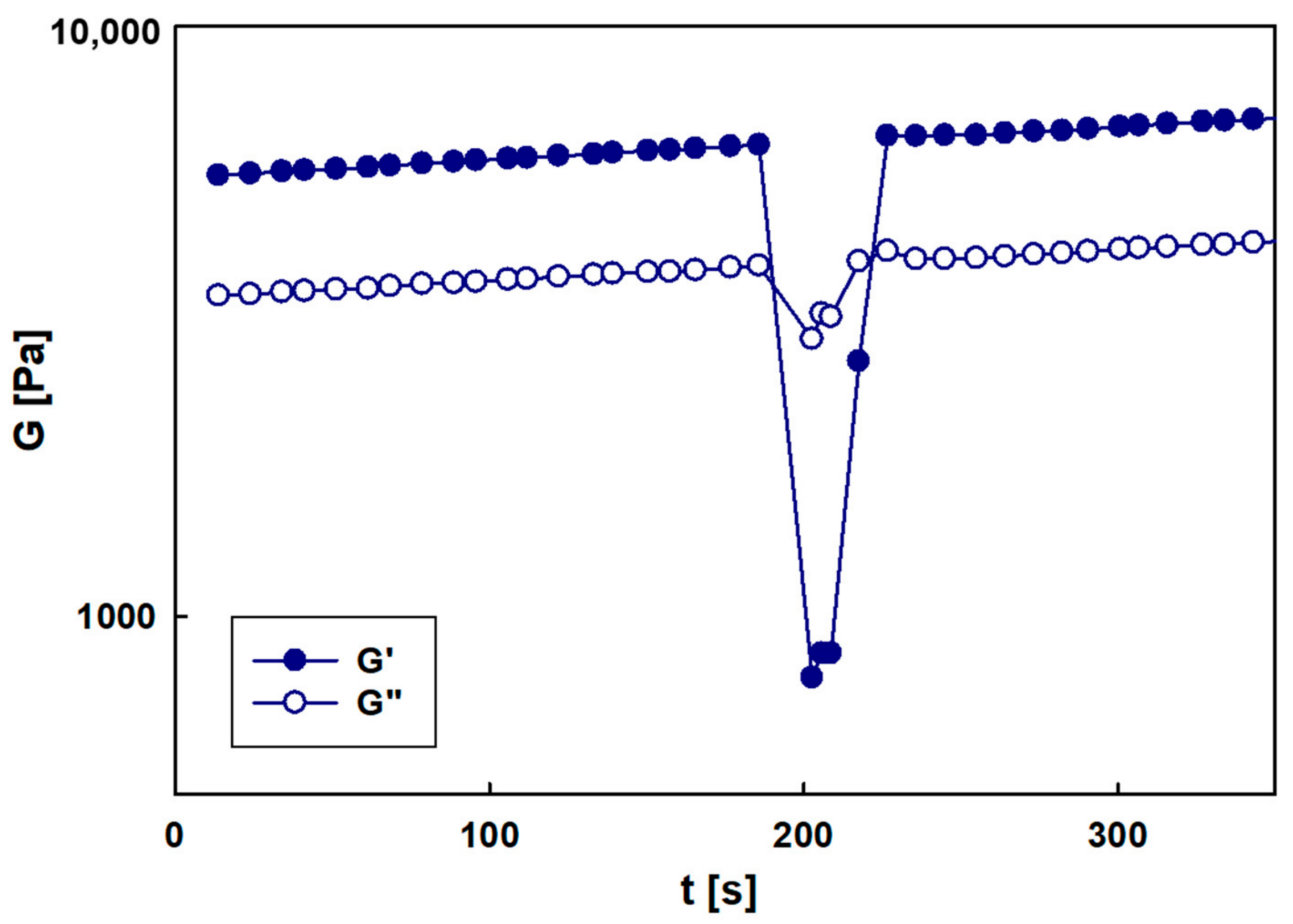
Time-dependent self-healing behaviour of the hydrogel H1.

The amplitude scan of PFGE-b-PGGE hydrogel with alternative γ = 1% and 300% demonstrated the immediate self-healing properties (Figure 8) thanks to continuous reformation of boronic ester cross-links in the polymer network. Increasing the strain value from 1% to 300% triggered the prompt drop of G′ from 6300 to 783 Pa, which was lower than G′′ (2990 Pa) and showed liquid-like behaviour. Upon the change of the strain to 1% again, the G′ value recovered to the initial value, indicating the self-healing properties of the investigated system thanks to the continuous rearrangement of boronic ester cross-links, which are in the equilibrium with boronic acid and 1,2-diol. These experiments demonstrate that the hydrogels presented here will form continuous layers and are well-adjusted to the surface layer and thus make them suitable for the vaginal treatment.

## 3. Materials and Methods

### 3.1. Materials

Furfuryl glycidyl ether, FGE (2,3-epoxypropyl 2-furylmethyl ether), d,l-isopropylideneglycerol (Solketal) and tetrabutyl ammonium bromide (TBAB) were purchased from Acros (Geel, Belgium). Epichlorohydrine and 60 wt% NaH in mineral oil was purchased from Merck (Merck, Darmstadt, Germany). Nifuratel was purchased from TCI. Tween 80 was purchased from ThermoScientific (Waltham, MA, USA).

### 3.2. Synthesis of (d,l-1,2-Isopropylidene glyceryl) Glycidyl Ether, IGG

IGG was synthesized according to Wurm’s procedure [46]. Briefly, 26 g (198 mmoL) of d,l-isopropylideneglycerol, Solketal was dissolved in 40 mL benzene, 40 mL of a 50% NaOH and 6.44 g (20 mmoL) tetrabutyl ammonium bromide TBAB was added. The mixture was cooled to approx. A temperature of 10 °C and 36.7 g (400 mmoL) of epichlorohydrine was slowly dropped. Then, the content was vigorously stirred at room temperature for 2 days, diluted with diethyl ether, washed three times with water, saturated solution of NaHCO_3_ and NaCl. The organic layer was dried over MgSO_4_. Finally, diethyl ether and an excess of epichlorohydrine was evaporated. The crude product was distilled under vacuum (10^−2^ mbar, 90 °C). The yield of the reaction was 50%.

^1^H NMR (200 MHz, DMSO-*d*_6_, δ, ppm): 4.1 (m, 1H), 3.9 (m, 1H), 3.1–3.7 (m, 6H), 3.05 (m, 1H), 2.7 (t, 1H), 2.5 (m, 1H), 1.2 (s, 3H), 1.3 (s, 3H)

### 3.3. Synthesis of Poly(furfuryl glycidyl ether), PFGE

A total of 0.166 g (1.24 mmoL) of 1,1,1-tris(hydroxymethyl)propane was dried by washing with 15 mL benzene, which was subsequently removed under vacuum. A total of 49.5 mg of 60 wt% NaH in mineral oil (eq. of 1.24 mmoL of NaH) was washed with 10 mL of anhydrous dioxane and dried under vacuum for 60 min. Then, NaH was added to 1,1,1-tris(hydroxymethyl)propane under argon and the mixture was stirred in dry THF in 45 °C for 24 h under vacuum. THF was evaporated and the alkoxide was dissolved in approx. 1 mL of distilled DMSO. A total of 11.4 g (74.24 mmoL) of FGE was distilled into the alkoxide. Subsequently, a fresh portion of dry THF (10 mL) was added. The polymerization was kept at 50 °C for 4 days under vacuum. The reaction was terminated by its exposure to air. The polymer was extracted in a water–dichloromethane mixture. The yield of the polymerization was 77.8%.

^1^H NMR (200 MHz, DMSO-*d*_6_, δ, ppm): 7.5 (s, 1H), 6.3 (s, 2H), 4.3 (s, 2H), 3.1–3.5 (br, 5H).

### 3.4. Synthesis of Poly(furfuryl glycidyl ether)-b-Poly((d,l-1,2-isopropylidene glyceryl) glycidyl ether), PFGE-b-PIGG

A total of 0.20 g (0.032 mmoL) of PFGE was dried by washing with benzene and it was evaporated under reduced pressure. A total of 2.5 mg of 60 wt% NaH in mineral oil (eq. of 0.064 mmoL of NaH) was washed with 10 mL of anhydrous dioxane and dried under reduced pressure for 1 h. Next, NaH was added to PFGE under argon and the mixture was stirred in dry THF at 45 °C for 24 h under vacuum.

Then, THF was evaporated and 2.6 mL (15.36 mmoL) of IGG was distilled in the reaction vessel. Dry THF was added to the mixture. The reaction was kept for 7 days at 70 °C under vacuum. The reaction was terminated by exposure of the mixture to air. The mixture was diluted with DMSO and dialyzed against DMSO using a dialysis tube (MWCO = 1 kDa), changing the dialysate four times. The yield of IGG polymerization was 88.7%.

^1^H NMR (200 MHz, DMSO-*d*_6_, δ, ppm): 7.5 (s, 1H), 6.3 (s, 2H), 4.3 (s, 2H), 4.1 (m, 1H), 3.9 (m, 1H), 3.2–3.7 (br, 12H), 1.1–1.4 (d, 6H).

### 3.5. Synthesis of Poly(furfuryl glycidyl ether)-b-Poly(glyceryl glycerol ether), PFGE-b-PGGE

A total of 0.688 g of PFGE-b-PIGG was dissolved in 10 mL of methanol. Subsequently, 0.2 mL of 1 M HCl_aq_ was added to the mixture. Th reaction was stirred at room temperature overnight. Then, 0.2 mL of 1 M aqueous solution of NaOH was added and methanol was evaporated under reduced pressure. The product was dialyzed against water and lyophilized.

^1^H NMR (200 MHz, DMSO-*d*_6_, δ, ppm): 7.5 (s, 1H), 6.3 (s, 2H), 4.6 (s, 1H), 4.5 (s, 1H), 4.3 (s, 2H), 3.1–3.7 (br, 15H).

### 3.6. Synthesis of Poly(2-acrylamidephenylboronic acid-ran-acrylamide), P(2-AAPBA-Ran-AM)

The copolymer was prepared by conventional radical polymerization initiated with AIBN by applying acrylamide (2 g; 28.10 mmoL) and 2-acrylamidophenylboronic acid pinacol ester (0.613 g; 2.24 mmoL). Polymerizations were carried out in 15 mL of DMF/dioxane mixture (5:1 *v*/*v*) at 70 °C. The synthesis was conducted for 16 h. The polymerization mixture was diluted in water, and the copolymer was precipitated into acetone and dried. Next, the copolymer was dissolved in an alkaline solution of NaOH (1 wt%) and dialyzed using a 1000 MW cut off dialysis membrane, at first against the alkaline aqueous solution and then against water, which was changed several times to reach the neutral pH. Dialysis was necessary to hydrolyze pinacol boronic esters and remove released pinacol. The copolymer was characterized using ^1^H NMR spectroscopy and GPC. The molar fraction of 2-AAPBA units in the copolymer 6 moL%, whereas M_n_ was 41000 (M_w_/M_n_ = 2.16).

### 3.7. Solubilization of Nifuratel within Poly(furfuryl glycidyl ether)-b-Poly(glyceryl glycerol ether)

A stock solution of nifuratel (0.41 mg/mL) in methanol was prepared. A total of 22.5 mg of PFGE-b-PGGE was dissolved in 2 mL of methanol. A total of 8 mL of nifuratel solution was added to the copolymer solution. The mixture was stirred for 30 min. Subsequently, methanol was allowed to evaporate at 30 °C overnight. The dry polymer-drug content was suspended in 2 mL of deionized water. The suspension was filtered twice through a 0.45 µm PTFE syringe filter. The aqueous solution was lyophilized overnight.

### 3.8. Drug Release

A total of 20 mg of drug-loaded copolymer was dissolved in 14 mL PBS pH = 7.4 and transferred into a regenerated cellulose dialysis membrane (MWCO = 3500). The dialysis bag was then immersed in 250 mL of PBS pH = 7.4 with 1% of Tween 80 (*v*/*v*). At given time points, 20 mL of the solution was collected and replaced with 20 mL of fresh PBS/Tween 80 solution. Subsequently, 3 × 45 mL of dichloromethane was added to each of the collected samples in order to extract nifuratel from the aqueous phase. The organic phases were liberated from dichloromethane by evaporation under reduced pressure. The dry product was dissolved in acetonitrile. The amount of nifuratel was calculated by measuring the absorbance at 361 nm and referring the results to the calibration curve prepared beforehand. The molar extinction coefficient of nifuratel in acetonitrile was ε = 16970 L moL^−1^ cm^−1^.

### 3.9. Instruments

#### 3.9.1. H NMR and ^1^H DOSY NMR Spectroscopy

Measurements were carried out at 295 K on a Bruker Avance 200 and Bruker Avance III 500 spectrometers (Karlsruhe, Germany). For ^1^H DOSY measurement, a sample was stabilized at the desired temperature for at least 10 min before data accumulation, and the 1 Hπ/2 pulse length was checked and adjusted carefully for each sample and temperature. The standard Bruker pulse programdstebpgp3s was selected for measurements using double stimulated echo for convection compensation and LED (Longitudinal Eddy Current Delay) using bipolar gradient pulses for diffusion and 3 spoil gradients. The shape of all gradient pulses was sinusoidal, the gradient spoil pulse was 0.6 ms, the delay for gradient recovery was set at 0.2 ms, and the LED was set at 5 ms and held constant in all experiments. The gradient pulse (small delta; δ/p30) was kept constant throughout.

#### 3.9.2. Gel Permeation Chromatography, GPC

The number average molecular weights (M_n_) of PFGE and PFGE-b-PIGG were determined by using an Agilent Pump 1100 Series (preceded by an Agilent G1379A Degasser), equipped with a set of two PLGel 5 μMIXED-C columns. A Wyatt Optilab Rex differential refractometer and Dawn Eos (Wyatt Technology Corporation, Santa Barbara, CA, USA) laser photometer were used as detectors. Dichloromethane was used as eluent at a flow rate of 0.8 mL min^−1^ at room temperature.

The number average molecular weights (M_n_) of PFGE-b-PGGE was determined by gel permeation chromatography (GPC) using a Shimadzu Pump LC-20AD and Shimadzu SIL*-20A* HT Autosampler. A refractometer RI-Optilab-T-rex-Wyatt and laser photometer DAWN 8+ (Wyatt Technology) were used as detectors. N,N′-dimethylformamide was used as eluent at a flow rate of 0.8 mL min^−1^ at 25 °C.

#### 3.9.3. Dynamic Light Scattering, DLS

The hydrodynamic diameter of micelles was measured at 298 and 310 K on Nano-ZS, Zetasizer (Malvern Panalytical, Warsaw, Poland).

#### 3.9.4. Transmission Electron Microscopy, TEM

TEM images were taken with Talos FX, FEI (Thermo Fisher Scientific, Waltham, MA USA). For TEM analysis, the aqueous solution of copolymer and the aqueous solution of copolymer saturated with nifuratel were, respectively, deposited on carbon-coated copper grid and evaporated. Image analysis was performed with Sigma Scan 5.0.

#### 3.9.5. Matrix-Assisted Laser Desorption/Ionization Time-of-Flight Mass Spectrometry, MALDI-TOF

MALDI-TOF spectrum of poly(furfuryl glycidyl ether) was recorded on MALDI-TOF/TOF Axima Performance Mass Spectrometer-Shimadzu Biotech. The polymer solution was prepared in CH_2_Cl_2_ at a concentration of 5 mg/L. Ditranol and potassium chloride were dissolved in THF at concentration 8 mg/L each. Then, 20 μL of polymer solution, 10 μL of ditranol and 1 μL of potassium salt were mixed together and evaporated. Mass spectra were obtained in the linear positive mode with an accelerating voltage of 5.6 mV. The number of scans was equal 200.

#### 3.9.6. Rheology

The rheological measurements of hydrogels were performed on a Thermoscientific HAAKE MARS 40 rheometer. A frequency sweep test (from 0.05 to 100 rad s^−1^ at 20 °C and 37 °C) was carried out using 8 mm plate-plate geometry using a 0.3 mm gap. A strain sweep for the hydrogel was performed at frequency 1 Hz in the range of strain from 0.02% to 600%.

The self-healing test was carried out in the 1 Hz oscillation time mode at 310 K by monitoring storage and loss moduli. First, the sample was placed under 1% of strain for 180 s, and then it was destroyed with a 5 s 300% strain pulse, after which 1% was again applied for sample restoration.

#### 3.9.7. Cytotoxicity Assessment

Human cervical cancer endothelial (HeLa) cells were grown in Dulbecco’s Modified Eagle Medium (DMEM). The 10% fetal bovine serum (FBS) and 1% streptomycin (100 mg·mL^−1^) were added to cell culture media. The cells were grown in T-75 culture flasks at 310 K in an atmosphere containing 5% CO_2_. The cells were sub-cultured every 2 or 3 days, then harvested and used in experiments after obtaining 80–90% confluence. The number of viable cells was determined by the trypan blue exclusion assay with the use of a Countess Automated Cell Counter (Invitrogen, Carlsbad, CA, USA). Cells were seeded in 96-well plates at 1.5 × 10^4^ cells/well in 100 µL of DMEM. After seeding, the plates were incubated for 24 h in a humidified atmosphere containing 5.0% CO_2_ at 310 K in order to allow cells to attach to the plates.

The influence of PFGE-b-PGG on cell viability was determined using the MTT-assay.

Briefly, to the 96-well plates containing cells at a density of 1.5 × 10^4^ cells/well, in medium different concentrations, PFGE-b-PGG were added. Cells were incubated with the copolymer for 24 and 48 h in a 310 K humidified atmosphere containing 5% CO_2_. After the incubation period cells were washed with 50 µL of phosphate buffered saline (PBS). Next, 50 µL of a 0.5 mg/mL solution of MTT in PBS was added to each well and cells were further incubated under normal culture conditions for 3 h. After incubation the residue MTT solution was removed and the obtained formazan precipitate was dissolved in DMSO (100 µL/well). The conversion of the tetrazolium salt (MTT) to a coloured formazan by mitochondrial and cytosolic dehydrogenases is a marker of cell viability. Before the absorbance measurement, plates were shaken for 1 min and the absorbance at 570 nm was measured on the PowerWave HT Microplate Spectrophotometer (BioTek, Winooski, VT, USA).

## 4. Conclusions

Two step ring-opening anionic polymerizations of epoxide comonomers, namely furfuryl glycidyl ether and (d,l-1,2-isopropylidene glyceryl) glycidyl ether followed by deprotection of diol groups, resulted in the formation of well-defined amphiphilic core-shell macromolecules that show no cytotoxicity. The furan-enriched core displayed high loading capacity and encapsulation efficiency of nifuratel. Therefore, this system proved to be a suitable solubilising agent for highly hydrophobic nifuratel. The numerous vicinal diol moieties present in the corona of PFGE-b-PGGE macromolecules assured the efficient cross-linking with 2-acrylamidephenylboronic acid statistically incorporated along the polyacrylamide, giving the viscoelastic hydrogel material. The hydrogel displayed injectable and self-healable properties, which along with the copolymer biocompatibility, makes it a highly promising platform in view of the gynaecological therapies of vulvovaginitis, and thus a good alternative to currently used suppositories.

## Figures and Tables

**Table 1:** The characteristics of synthesized polymers.

Polymer	DP_n_ FGE (^1^H NMR)	DP_n_ GGE (^1^H NMR)	M_n_ (^1^H NMR)	M_n_ (GPC)	M_w_/M_n_ (GPC)
PFGE	39	-	6000	4930	1.11
PFGE-b-PGGE	39	426	61,000	57,800	1.28

**Table 2:** The composition of hydrogel systems based on PFGE-b-PGGE cross-linked with boronic-acid-based polyacrylamide.

Hydrogel	PFGE-b-PGGE (g)	P(2-AAPBA-b-AM) (g)	Weight Polymer Fraction (wt%)
H1	0.055	0.049	23
H2	0.055	0.025	18.5

## Data Availability

The data sets used and/or analyzed within the frame of the study can be provided by the corresponding author upon reasonable request.

## Supplementary Materials

The following are available online at https://www.mdpi.com/article/10.3390/ijms22168386/s1, Figure S1.: ^1^H NMR spectrum of furfuryl glycidyl ether, FGE recorded in DMSO-*d*_6_, Figure S2: ^1^H NMR spectrum of (d,l-1,2-isopropylidene glyceryl) glycidyl ether, IGG recorded in DMSO-*d*_6_, Figure S3; ^1^H DOSY NMR spectrum of PFGE-b-PIGG recorded in DMSO-*d*_6_, Figure S4: ^1^H NMR spectrum of nifuratel recorded in DMSO-*d*_6_, Figure S5: ^1^H NMR spectrum of PFGE-b-PGGE saturated with nifuratel recorded in DMSO-*d*_6_. The spectrum was recorded for the sample after the process of encapsulation in methanol, methanol evaporation, and then the sample suspension in deionized water, filtration via 0.45 µm PTFE filter, and lyophilization, Figure S6: The comparison of aqueous solution of nifuratel (a) and nifuratel-enriched-PFGE-b-PGGE macromolecules (b), Figure S7: The dependence of absorbance on molar concentration of nifuratel, Figure S8: ^1^H NMR spectrum of P(2-AAPBA-ran-AM) recorded in D_2_O.
