# Supplementary material for: Star-Shaped Poly(furfuryl glycidyl ether)-Block-Poly(glyceryl glycerol ether) as an Efficient Agent for the Enhancement of Nifuratel Solubility and for the Formation of Injectable and Self-Healable Hydrogel Platforms for the Gynaecological Therapies
Source: Int J Mol Sci. 2021 Aug 4;22(16):8386. doi: 10.3390/ijms22168386 (PMC8395068; doi:10.3390/ijms22168386)
Supplement: Supplementary file 1 [file ijms-22-08386-s001.zip › ijms-1305457-supplementary.pdf]

Star-shaped poly(furfuryl glycidyl ether)-block-poly(glyceryl glycerol) as an efficient agent for the enhancement of nifuratel solubility and for the formation of injectable and self-healable hydrogel platforms for the gynaecological therapies

Piotr Ziemczonek, Monika Gosecka*, Mateusz Gosecki, Monika Marcinkowska, Anna Janaszewska, Barbara Klajnert-Maculewicz

Centre of Molecular and Macromolecular Studies, Polish Academy of Sciences
Sienkiewicza 112, 90-363 Lodz, Poland
* Correspondence: mdybko@cbmm.lodz.pl

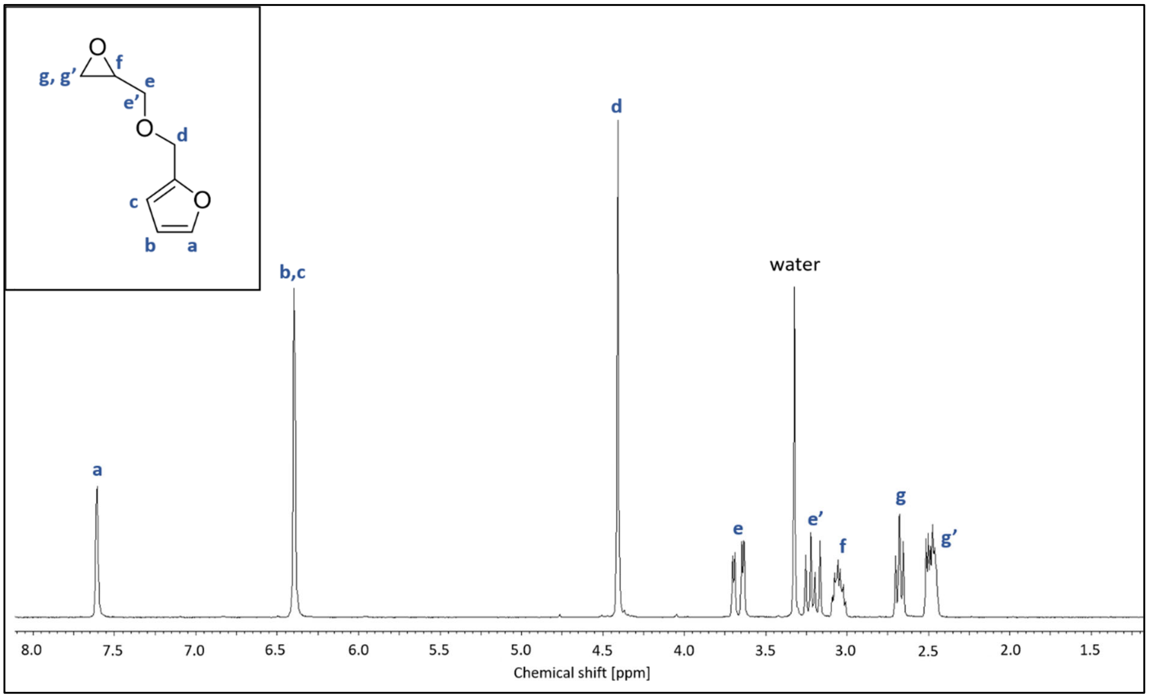

Figure S1. ^{1}H NMR spectrum of furfuryl glycidyl ether, FGE recorded in DMSO-d_6.

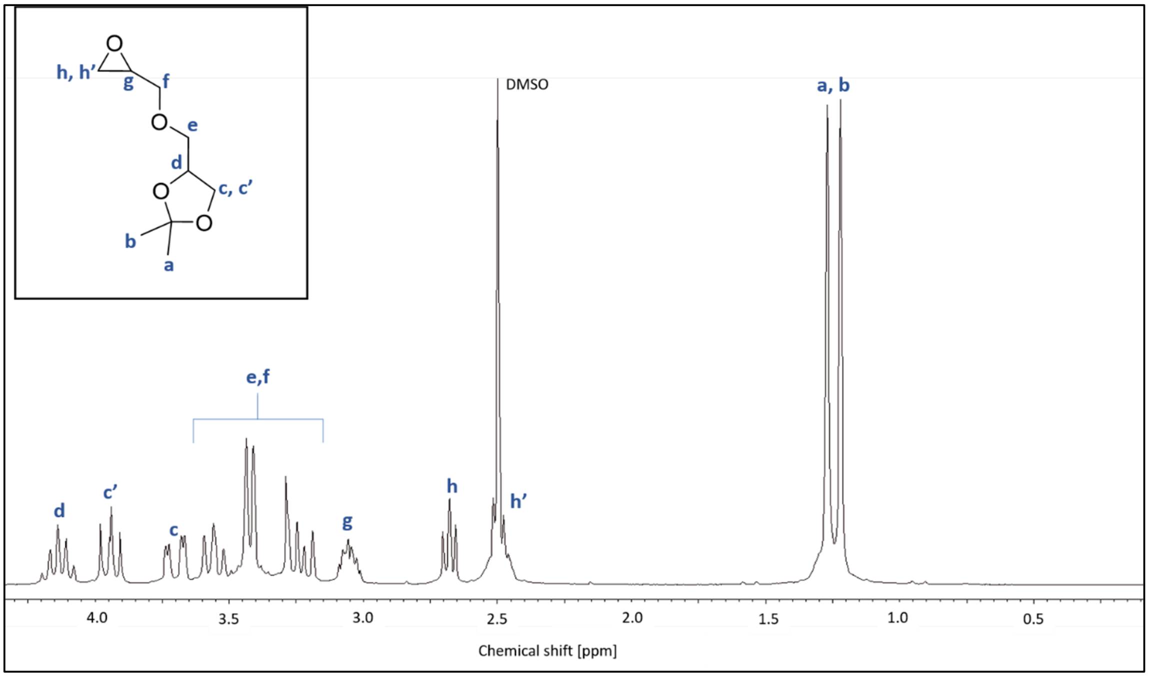

Figure S2. ^{1}H NMR spectrum of (D,L-1,2-isopropylidene glyceryl) glycidyl ether, IGG recorded in DMSO-d_6.

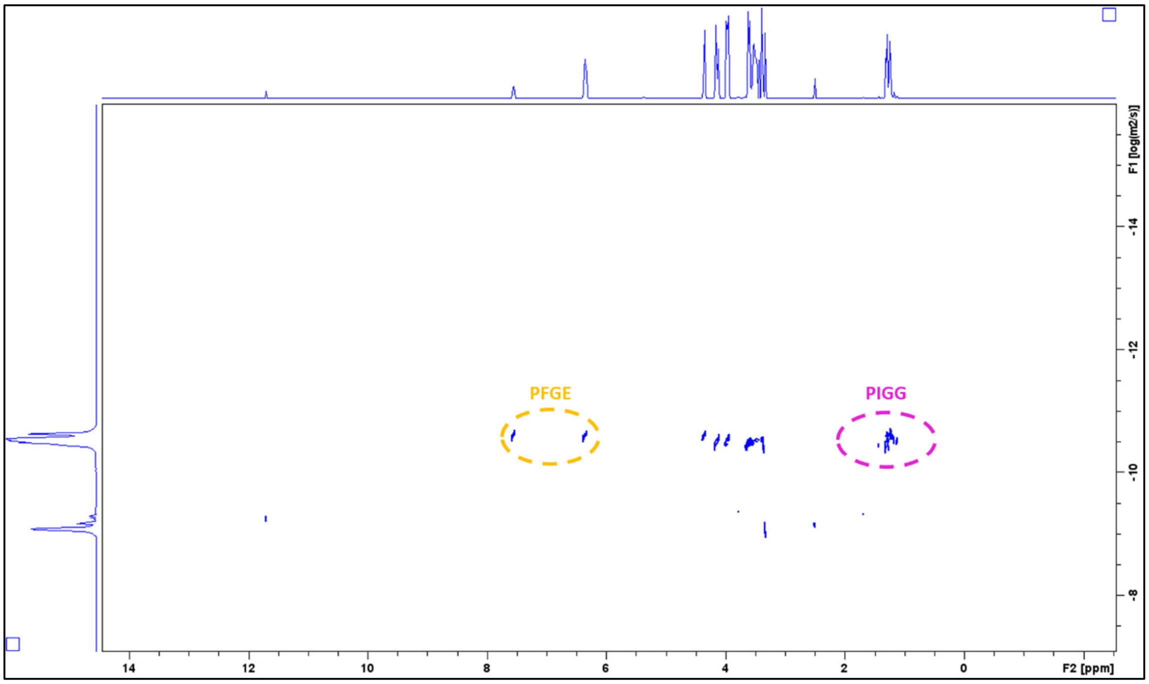

Figure S3. ^{1}H DOSY NMR spectrum of PFGE-b-PIGG recorded in DMSO-d_6.

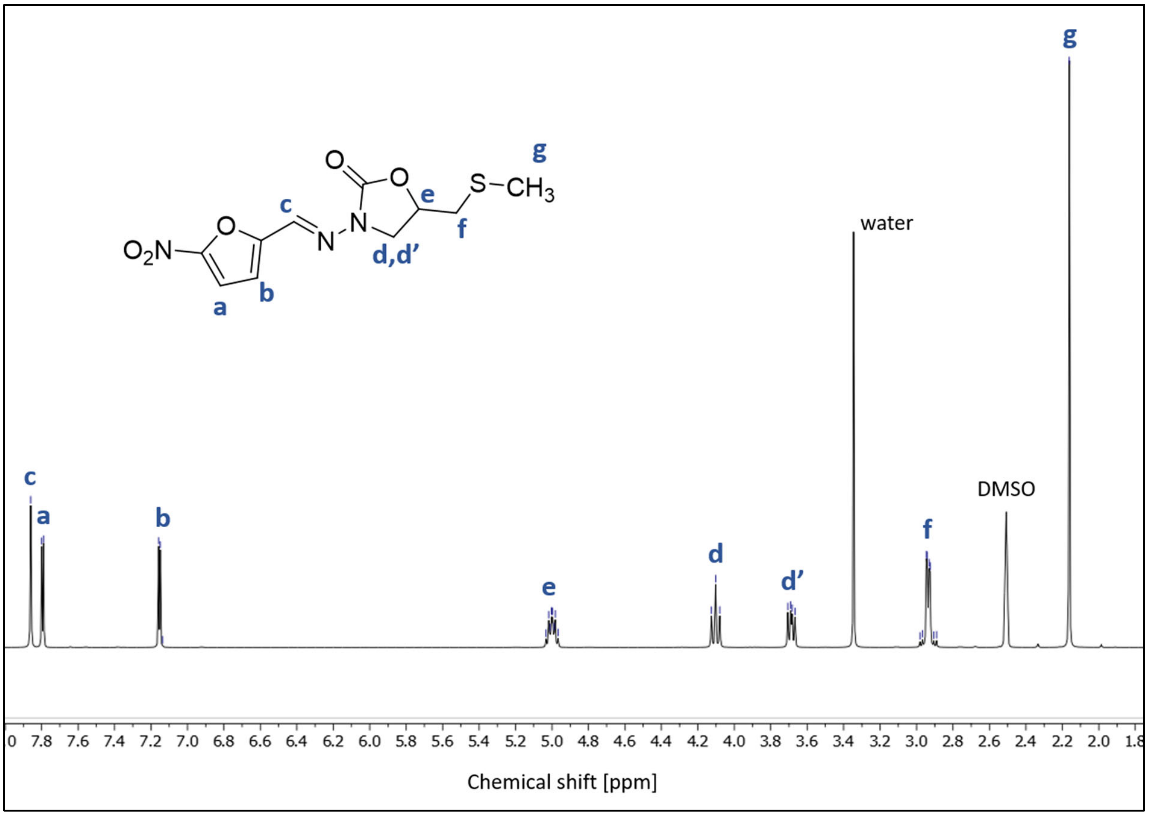

Figure S4. ^{1}H NMR spectrum of nifuratel recorded in DMSO-d_6.

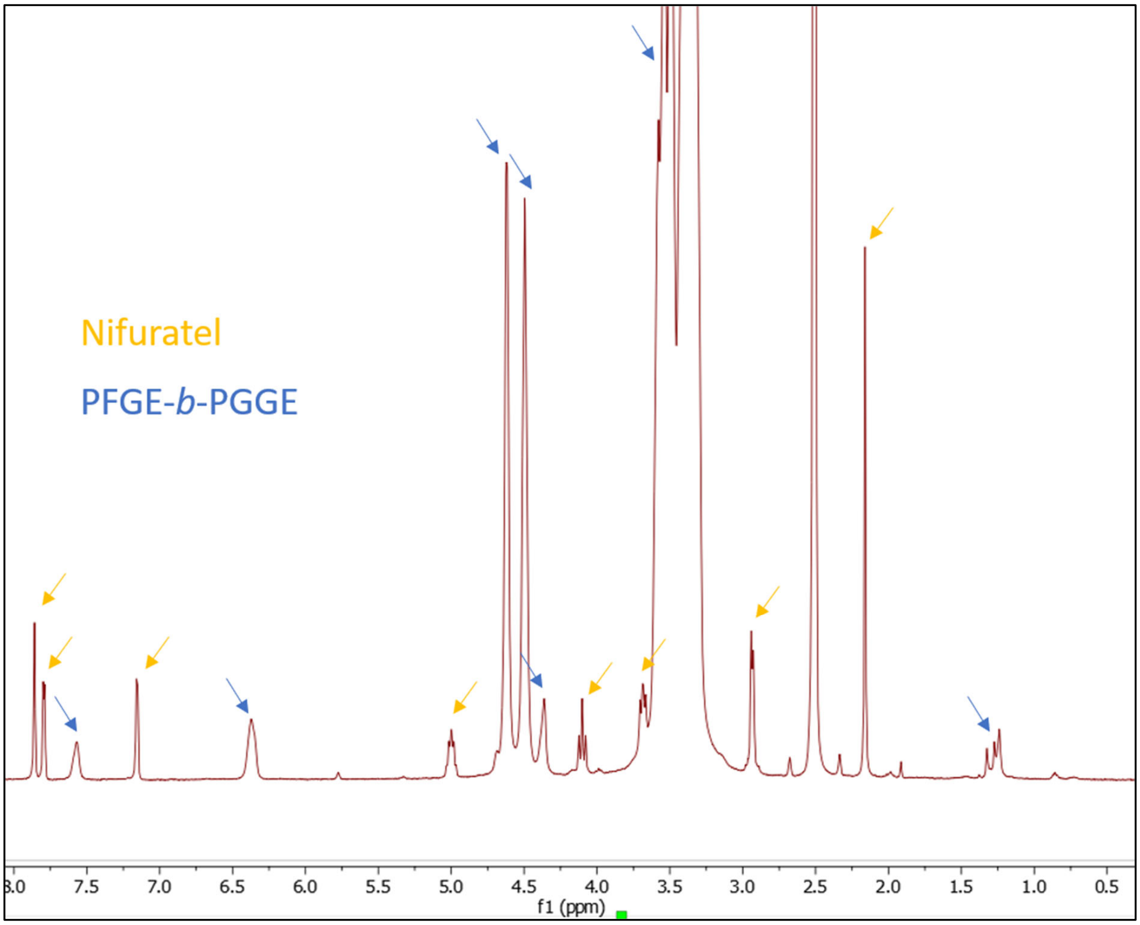

Figure S5 ^{1}H NMR spectrum of PFGE-b-PGGE saturated with nifuratel recorded in DMSO-d_6. The spectrum was recorded for the sample after the process of encapsulation in methanol, methanol evaporation, and then the sample suspension in deionized water, filtration via 0.45 µm PTFE filter, and lyophilization.

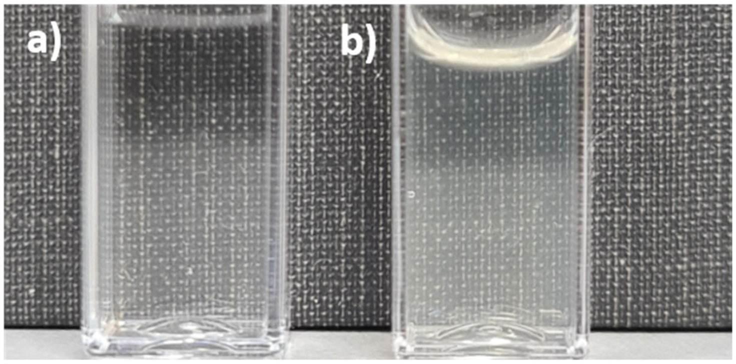

Figure S6. The comparison of aqueous solution of nifuratel (a) and nifuratel-enriched-PFGE-b-PGGE macromolecules (b).

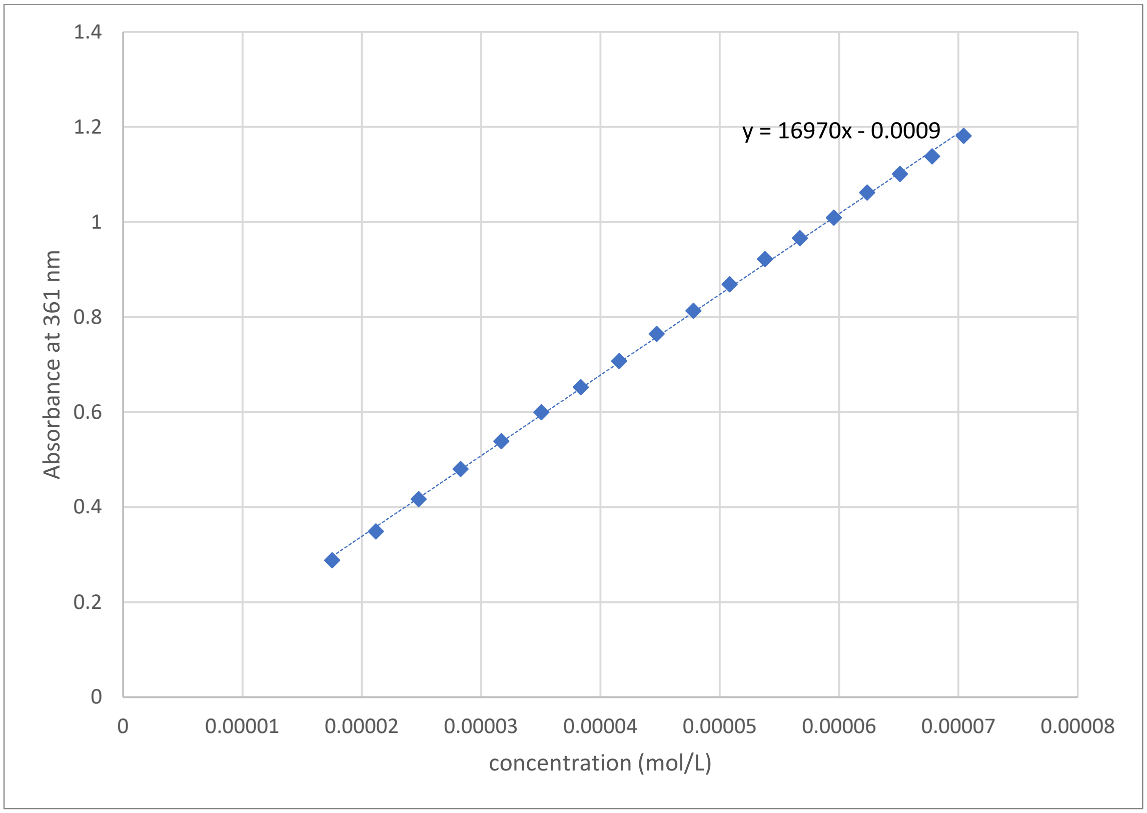

Figure S7. The dependence of absorbance on molar concentration of nifuratel.

Molar extinction coefficient (ε) of nifuratel was calculated using Lambert-Beer equation according to experimental results obtained for a series of nifuratel solutions at different molar concentration in acetonitrile. The maximum absorbance of nifuratel was found at λ_{max} = 361 nm.

$$A = \varepsilon \ell c$$

where:

A - absorbance

ε -molar extinction coefficient

ℓ - path length

c - molar concentration

ε (nifuratel) = 16970 L $mol^{-1}cm^{-1}$

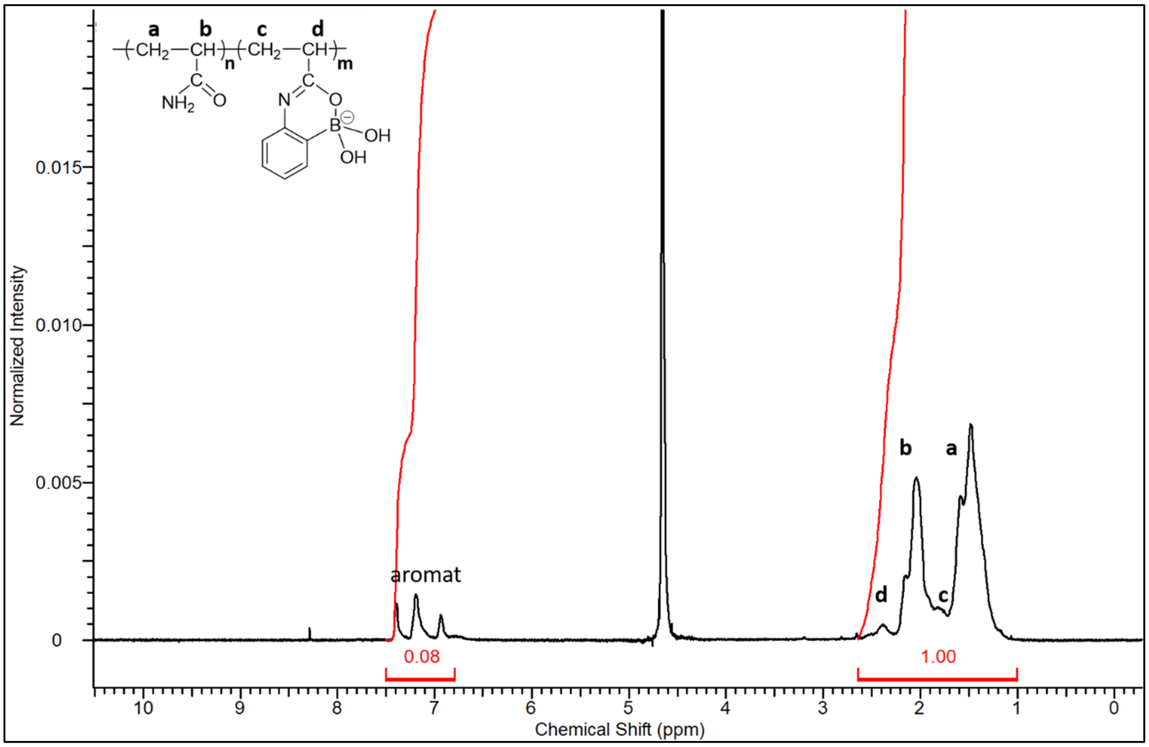

Figure S8. ^{1}H NMR spectrum of P(2-AAPBA-ran-AM) recorded in D_2O.
